# Associations Between Maladaptive Perfectionism and Life Satisfaction Among Chinese Undergraduate Medical Students: The Mediating Role of Academic Burnout and the Moderating Role of Self-Esteem

**DOI:** 10.3389/fpsyg.2021.774622

**Published:** 2022-01-07

**Authors:** Qinghua Wang, Huazhang Wu

**Affiliations:** ^1^Institute of Foreign Languages, China Medical University, Shenyang, China; ^2^Department of Health Service Administration, College of Health Management, China Medical University, Shenyang, China

**Keywords:** maladaptive perfectionism, life satisfaction, academic burnout, self-esteem, moderated mediation, medical students

## Abstract

Empirical research has shown that maladaptive perfectionism may lead to lower life satisfaction. However, the relationship between maladaptive perfectionism and life satisfaction among medical students and the mechanism underlying this relationship still need to be further explored. The present study used a large sample of undergraduate medical students to examine the associations between maladaptive perfectionism and life satisfaction and to explore the mechanism underlying the associations. Specifically, the present study tried to probe the mediating role of academic burnout and the moderating role of self-esteem in the relationship between maladaptive perfectionism and life satisfaction in medical students. We invited 1628 undergraduate medical students from two medical universities in Northeastern China to participate in the survey. Among the students recruited, 1377 medical students (response rate: 84.6%) completed questionnaires including the Frost Multidimensional Perfectionism Scale (FMPS) maladaptive perfectionism subscales, the Chinese College Student Academic Burnout Inventory (CCSABI), the Satisfaction With Life Scale (SWLS), the Rosenberg Self-Esteem Scale (RSES) and demographic information. Results show that maladaptive perfectionism was significantly negatively related to life satisfaction among medical students and academic burnout played a significant mediating role (β = −0.10, BCa 95%CI: −0.12, −0.07) in this relationship. Moderated mediation analyses reveal that the mediating effect of maladaptive perfectionism on life satisfaction *via* academic burnout was moderated by self-esteem. Maladaptive perfectionism exerted a stronger effect on life satisfaction *via* the mediating role of academic burnout for medical students with high self-esteem [β = −0.026, SE = 0.009, 95%CI = (−0.047, −0.011)] than for medical students with low self-esteem [β = −0.019, SE = 0.009, 95%CI = (−0.038, −0.001)]. Medical institutions can implement effective interventions to decrease medical students’ maladaptive perfectionism levels and academic burnout levels, and increase their self-esteem levels in order to enhance their life satisfaction.

## Introduction

Medical students’ mental health has long been a focus of research in medical education and literature shows that the mental state of medical students has a considerable impact on their academic performance (Kötter et al., 2017), social relationships (Arbabisarjou et al., 2016) and life quality (Lucchetti et al., 2018). Since the mental health of medical students would affect the quality of healthcare they provide as physicians in the future, it is of vital importance to revisit the hot topic.

Research reveals that perfectionism as a personality trait among medical students exerts a significant influence on their mental health (Seeliger and Harendza, 2017; Bußenius and Harendza, 2019). Two categories of perfectionism have been identified: adaptive perfectionism and maladaptive perfectionism (Rice and Ashby, 2007). Adaptive perfectionism is characterized by striving toward high personal standards without criticizing oneself if the standards are not met, while maladaptive perfectionism features being extremely self-critical when goals are not achieved (Bieling et al., 2004). Studies show that adaptive perfectionism is associated with positive outcomes, such as life satisfaction, adaptive coping styles and positive affect (Rice and Mirzadeh, 2000; Rice and Lapsley, 2001; Ashby et al., 2012). In contrast, maladaptive perfectionism has been found to be correlated with psychological dysfunctions, such as depression, negative affect and hopelessness (Dunkley et al., 2003; Rice et al., 2006; Bergman et al., 2007).

As an important mental health indicator, life satisfaction entails an individual’s subjective evaluation of the life as a whole which is based on the individual’s self-set standards (Diener et al., 1985). Although some cross-sectional studies have demonstrated the negative relationship between maladaptive perfectionism and life satisfaction among different population groups such as undergraduate students (Ashby et al., 2012; Gnilka et al., 2013; Chen et al., 2017), high school adolescents (Ongen, 2009), Internet users (Stoeber and Stoeber, 2009) and career mothers (Mitchelson and Burns, 1998), we found few studies exploring the association of maladaptive perfectionism with life satisfaction among medical students and the underlying mechanism in this association. Therefore, the current research aimed to examine the relationship between maladaptive perfectionism and life satisfaction with a large sample of undergraduate medical students and to further explore the underlying mechanism in this relationship. Based on the results of the association between maladaptive perfectionism and life satisfaction in previous studies (Mitchelson and Burns, 1998; Ongen, 2009; Stoeber and Stoeber, 2009; Ashby et al., 2012; Gnilka et al., 2013; Chen et al., 2017), we hypothesized that the relationship between maladaptive perfectionism and life satisfaction in medical students was negative and the first aim of the current research was to test this hypothesis.

Literature shows that maladaptive perfectionism was significantly positively correlated with academic burnout in medical students (Yu et al., 2016). The concept of “burnout” was first put forward by Freudenberger in 1974, which specifically refers to “staff burnout” in the occupational field (Freudenberger, 1974). Maslach et al. (1996) defined “job burnout” as a psychological syndrome characterized by emotional exhaustion (lack of energy and enthusiasm toward work, feeling fatigue), depersonalization (indifferent to and deliberately keeping a distance from the object of work) and a feeling of professional inefficacy (feeling low self-worth and a reduced sense of achievement at work). Based on the concept of “job burnout” in the work environment experienced by employees, Lian and colleagues developed the concept of “academic burnout” in the learning environment experienced by students (Lian et al., 2005). “Academic burnout”, also known as “learning burnout” or “school burnout”, is a kind of burnout syndrome characterized by prolonged negative emotions (lack of interest toward study, feeling bored and frustrated), improper learning behaviors (unplanned and passive learning behaviors due to low initiative and low motivation in study) and low achievement (feeling incompetent to deal with learning requirements and a reduced sense of academic achievement). Although research shows that academic burnout can negatively predict life satisfaction in medical students (Wang et al., 2019), no study has yet examined the mediating role of academic burnout in the relationship between maladaptive perfectionism and life satisfaction in this population group. Thus, the second aim of the current research was to test this mediation.

Self-esteem involves how we view and feel about ourselves, which exerts a profound influence on how we live (Rosenberg, 1965). In Maslow’s hierarchy of needs, self-esteem is placed at the second from the top of the pyramid. According to Maslow, individuals are not able to develop or obtain self-actualization without satisfying the need for self-esteem (Maslow, 1943). According to the JD-R model presented by Demerouti et al. (2001), in the work environment, if the demands of the job exceed the resources an employee possesses, he may feel incompetent to meet the job requirements and stresses out, and if this stressful situation continues for an extended period of time, it is likely that the employee feels depleted and burned out toward work in the end. Similarly, in the learning environment, if the academic demands exceed the resources a student possesses, he may feel incapable of meeting the learning requirements and if the sense of this perceived incapability prolongs, academic burnout may finally set in. Self-esteem is an important psychological resource and has been demonstrated to be a mental health protector against academic burnout (Luo et al., 2016). In addition, empirical research has also confirmed the positive association of self-esteem with life satisfaction (Kupcewicz et al., 2020) and the negative correlation of self-esteem with maladaptive perfectionism (Mirzairad et al., 2017). Therefore, we hypothesized that self-esteem, as a moderator, may affect the mediating role of academic burnout in the relationship between maladaptive perfectionism and life satisfaction among medical students. Thus, the third aim of the current research was to test this moderated mediation model.

**FIGURE 1:**
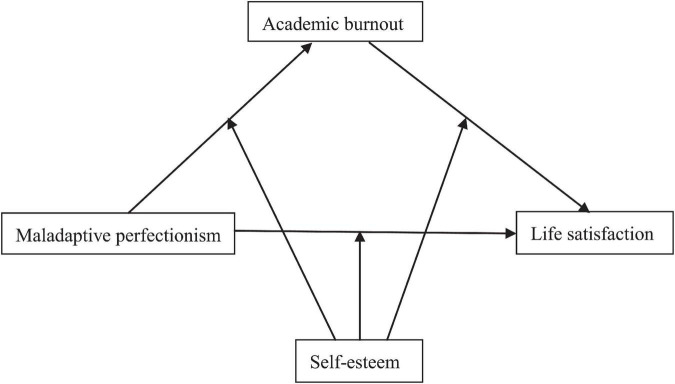
The conceptual model of the current research.

To sum up, the current research tried to examine the association between maladaptive perfectionism and life satisfaction among medical students and to probe the underlying mechanism in this association. Specifically, the current research aimed to explore the mediating role of academic burnout in the relationship between maladaptive perfectionism and life satisfaction and the moderating role of self-esteem in this relationship with a large sample of Chinese undergraduate medical students. The conceptual model of the current research was presented in Figure 1.

## Materials and Methods

### Participants

The participants in the current research were 1377 undergraduate medical students from two medical universities in Northeastern China. As for the demographic characteristics of the participants, 501 (36.4%) were male and 876 (63.6%) were female with the age ranging from 17 to 24 years old (mean: 19.44, standard deviation: 1.35); 437 (31.7%) were freshmen, 382 (27.8%) were sophomore, 280 (20.3%) were juniors and 278 (20.2%) were seniors; 809 (58.8%) were the only child and 568 (41.2%) were not the only child in the family; 668 (48.5%) came from cities, 335 (24.3%) from towns and 374 (27.2%) from villages.

### Procedure

This cross-sectional study was conducted at two medical universities in Northeastern China. A random cluster sampling method was adopted, and online questionnaires were distributed from October to November 2019 to whole classes of medical students in their 1st to 4th academic years. An online survey platform called Juanxing Wen was used to collect data for this study. The purpose of the study (i.e., for research purposes only) was explained beforehand by trained research investigators. Students were assured that participation was voluntary and the data collected would be kept confidential. Among 1628 medical students recruited for this study, 1377 students (response rate 84.6%) agreed to participate. It took about 15 min to complete the online questionnaire and every participant signed the informed consent form online. The participants finally included in the present study met the following criteria: (1) are full-time undergraduate medical students; (2) are proficient in Chinese; (3) are willing to participate. Students who are not full-time undergraduate medical students, who are international medical students not proficient in Chinese and who disagreed to participate were excluded. The study was approved by the Institutional Review Board of the authors’ affiliated medical university and was conducted according to the Declaration of Helsinki (59th WMA General Assembly, 2008).

### Measures

#### Maladaptive Perfectionism

The Frost Multidimensional Perfectionism Scale (Frost et al., 1990) maladaptive perfectionism subscales were used to assess medical students’ levels of maladaptive perfectionism. The original English version of the Frost Multidimensional Perfectionism Scale (FMPS) included four maladaptive perfectionism subscales: Concern over Mistakes (9 items), Parental Expectations (5 items), Doubts about Actions (4 items) and Parental Criticism (5 items). However, the Chinese version of FMPS supported the five-factor construct without the maladaptive perfectionism subscale of Parental Criticism (Cheng et al., 1999), so we did not include this subscale in the present study. Zi tested the Chinese version of the maladaptive perfectionism subscales and reduced the 9 items of the Concern over Mistakes subscale in the original English version to 6 items, which subsequently demonstrated good reliability in Chinese college students (Zi, 2007). Therefore, the three maladaptive perfectionism subscales of FMPS used in the present study were: Concern over Mistakes (6 items), Parental Expectations (5 items) and Doubts about Actions (4 items). Each item was scored on a 5-point Likert scale, ranging from 1 (completely disagree) to 5 (completely agree), and the total score was calculated by summing up the score for each maladaptive perfectionism subscale with a higher total score indicating a higher level of maladaptive perfectionism. The Cronbach’s alpha coefficients for Concern over Mistakes, Parental Expectations and Doubts about Actions in the present study were 0.866, 0.776, and 0.655, respectively, and the Cronbach’s alpha coefficient for the total maladaptive perfectionism scale was 0.852.

#### Academic Burnout

Medical students’ academic burnout levels were measured by the Chinese College Student Academic Burnout Inventory (CCSABI) developed by Lian et al. (2005), which is based on Maslach’s Occupational Burnout Inventory (Maslach et al., 1996). There were a total of 20 items falling into three subscales for this Academic Burnout Inventory: Low Mood (8 items), Improper Behavior (6 items) and Low Achievement (6 items). Each item was scored on a 5-point Likert scale from 1 (not true of me at all) to 5 (very true of me). After the negatively worded questions were reverse scored, the sum score was calculated with a higher sum score indicating a higher level of academic burnout. CCSABI has been widely used in China among college students and has demonstrated good psychometric properties in Chinese medical students (Wang and Du, 2020). The Cronbach’s alpha coefficients for the three subscales of the inventory were: Low Mood (α = 0.832), Improper Behavior (α = 0.704) and Low Achievement (α = 0.689), respectively. The Cronbach’s alpha coefficient for the whole inventory in the present study was 0.875.

#### Life Satisfaction

Medical students’ life satisfaction levels were measured by the Satisfaction With Life Scale (SWLS) developed by Diener et al. (1985). This uni-dimensional scale included five items scored from 1 “strongly disagree” to 7 “strongly agree” with a higher total score indicating a higher life satisfaction level. SWLS has shown good reliability among Chinese medical students in previous research (Wang et al., 2019). The Cronbach’s alpha coefficient for SWLS was 0.882 in the present study.

#### Self-Esteem

The 10-item Rosenberg Self-Esteem Scale (RSES; Rosenberg, 1965) was adopted to measure medical students’ global self-esteem. Each item was scored on a 4-point Likert scale, ranging from 1 (not true of me at all) to 4 (very true of me). After the scores for negatively worded questions were reversed, the total score was calculated to indicate the overall level of medical students’ self-esteem. The Chinese version of this scale has demonstrated sufficient reliability (Yu et al., 2019), and the Cronbach’s alpha coefficient of RSES in the present study was 0.871.

### Data Analyses

Statistical Packages for Social Sciences (SPSS) version 22.0 was used for descriptive statistics analysis of participants’ socio-demographic characteristics, reliability analysis of measures, and Pearson’s correlation analysis among the studied psychological variables. The macro program PROCESS of SPSS (Hayes, 2013) was employed for conducting the moderated mediation analysis. For this analysis, Model 59 in the macro program PROCESS (version 3.3) was adopted. Maladaptive perfectionism was entered as the independent variable, life satisfaction as the dependent variable, academic burnout as the mediator, self-esteem as the moderator and socio-demographic characteristics of students’ gender, age, grade, the only child or not and their hometowns were entered as covariates. The conditional direct and indirect effects were estimated by using asymptotic and resampling strategies developed by Preacher and Hayes (2008) with 5000 bootstrap samples. The effects were considered significant when the bias-corrected and accelerated 95% confidence interval (BCa 95%CI) did not include zero. Two-tailed *p* values smaller than 0.05 were considered as statistically significant.

## Results

### Correlations Among Maladaptive Perfectionism, Academic Burnout, Life Satisfaction and Self-Esteem

Means, standard deviations, and Pearson correlation coefficients of maladaptive perfectionism, academic burnout, life satisfaction and self-esteem among medical students are presented in Table 1. The results show that all correlation coefficients were significant (*p* < 0.01) and in the expected directions. Specifically, maladaptive perfectionism was significantly positively associated with academic burnout (*r* = 0.31, *p* < 0.01), while significantly negatively associated with life satisfaction (*r* = −0.25, *p* < 0.01) and self-esteem (*r* = −0.38, *p* < 0.01). Academic burnout was significantly negatively correlated with both life satisfaction (*r* = −0.36, *p* < 0.01) and self-esteem (*r* = −0.49, *p* < 0.01). The relationship between life satisfaction and self-esteem was significantly positive (*r* = 0.43, *p* < 0.01) among medical students.

**TABLE 1:** Means, standard deviation (SD) and correlations of psychological variables.

Variables	Mean	SD	1	2	3	4
1. Maladaptive perfectionism	40.54	10.94	^1^			
2. Academic burnout	52.85	12.08	0.31**	^1^		
3. Life satisfaction	22.57	6.64	−0.25**	−0.36**	^1^	
4. Self-esteem	30.95	4.81	−0.38**	−0.49**	0.43**	^1^

N=1377; **p<0.01 (two-tailed).

### Testing for the Mediation Model

As shown in Table 2, after controlling for demographic variables of gender, age, grade, only child or not and residence, the total effect of maladaptive perfectionism on life satisfaction was significantly negative (β = −0.27, *t* = −10.20, *p* < 0.001). Meanwhile, maladaptive perfectionism was significantly positively associated with academic burnout (β = 0.31, *t* = 11.98, *p* < 0.001), which in turn was significantly negatively associated with life satisfaction (β = −0.30, *t* = −11.70, *p* < 0.001). After adding academic burnout as a mediator in the model, the direct effect of maladaptive perfectionism on life satisfaction was still significantly negative (β = −0.17, *t* = −6.57, *p* < 0.001). The mediation analysis with 5000 bootstrap samples shows that the indirect effect of maladaptive perfectionism on life satisfaction *via* academic burnout was −0.10, and since its bias-corrected and accelerated 95% confidence interval (BCa 95%CI) did not include 0 (BCa 95%CI: −0.12, −0.07), academic burnout partially mediated the relationship between maladaptive perfectionism and life satisfaction (Hayes, 2013). The mediation effect of academic burnout accounted for 37% of the total effect of maladaptive perfectionism on life satisfaction among medical students.

**TABLE 2:** The mediation analysis.

Predictors (IV)	Model1 (DV: LS)		Model2 (DV: AB)		Model3 (DV: LS)	
	
	β	*t*	β	*t*	β	*t*
Gender	0.02	0.77	0.00	0.10	0.02	0.84
Age	–0.15	−2.94**	0.06	1.10	–0.13	−2.73**
Grade	0.04	0.78	–0.01	–0.27	0.04	0.73
Only child	–0.06	−2.13*	0.01	0.23	–0.06	−2.16*
Residence	–0.13	−4.47***	0.06	1.84	–0.12	−4.11***
MP	–0.27	−10.20***	0.31	11.98***	–0.17	−6.57***
AB					–0.30	−11.70***
*R* ^2^	0.11		0.10		0.19	
*F*	27.31***		25.02***		45.28***	

N = 1377; IV, independent variable; DV, dependent variable; MP, maladaptive perfectionism; AB, academic burnout; LS, life satisfaction. Gender (1 = male, 2 = female); Grade (1 = freshman, 2 = sophomore, 3 = junior, 4 = senior); Only child (1 = only child, 2 = not only child); Residence (1 = cities, 2 = towns, 3 = villages). *p < 0.05 (two-tailed), **p < 0.01 (two-tailed), ***p < 0.001 (two-tailed).

### Testing for the Moderated Mediation Model

**FIGURE 2:**
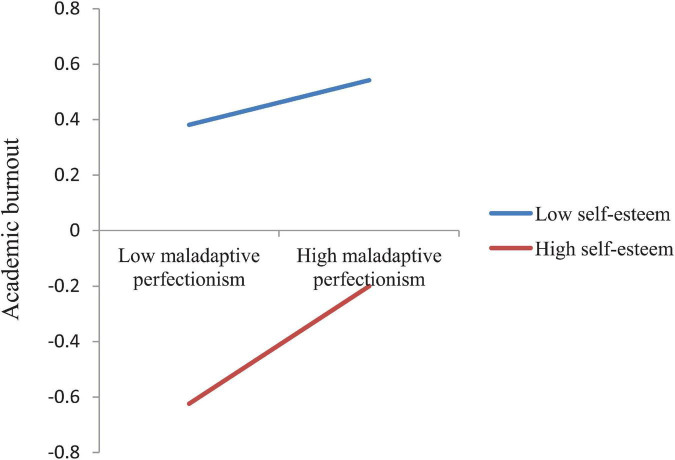
The interaction effect of maladaptive perfectionism and self-esteem on academic burnout.

The moderated mediation model was tested by using Model 59 in the macro program PROCESS of SPSS (Hayes, 2013) and the results are shown in Table 3. As can be seen from the table, in Model 1 with academic burnout as the dependent variable, after controlling for socio-demographic variables (gender, age, grade, only child, residence), maladaptive perfectionism was significantly positively correlated with academic burnout (β = 0.15, *t* = 5.73, *p* < 0.001), while self-esteem was significantly negatively associated with academic burnout (β = −0.44, *t* = −17.32, *p* < 0.001). The interaction effect of maladaptive perfectionism and self-esteem (Maladaptive perfectionism × Self-esteem) on academic burnout was significant (β = 0.07, *t* = 3.03, *p* < 0.01). We plotted this interaction effect at two levels of self-esteem (1 SD below the mean and 1SD above the mean) in Figure 2. Simple slope tests show that the association between maladaptive perfectionism and academic burnout was stronger for medical students with high self-esteem (β*_*simple*_* = 0.21, *t* = 6.30, *p* < 0.001) than for medical students with low self-esteem (β*_*simple*_* = 0.08, *t* = 2.41, *p* < 0.05).

**TABLE 3:** The moderated mediation analysis.

	β	*t*	*p*
**Model 1: Dependent variable: academic burnout**			
Gender	0.00	0.05	0.96
Age	0.02	0.50	0.62
Grade	0.00	0.10	0.92
Only child	0.00	0.14	0.89
Residence	0.02	0.80	0.43
Maladaptive perfectionism	0.15	5.73	<0.001
Self-esteem	–0.44	–17.32	<0.001
Maladaptive perfectionism × Self-esteem	0.07	3.03	<0.01
*R* ^2^	0.26		
*F*	60.98***		
**Model 2: Dependent variable: life satisfaction**			
Gender	0.02	0.87	0.38
Age	–0.11	–2.44	<0.05
Grade	0.03	0.60	0.55
Only child	–0.06	–2.15	<0.05
Residence	–0.10	–3.78	<0.001
Maladaptive perfectionism	–0.10	–3.66	<0.001
Academic burnout	–0.18	–6.60	<0.001
Self-esteem	0.31	10.90	<0.001
Maladaptive perfectionism × Self-esteem	–0.06	–2.45	<0.05
Academic burnout × Self-esteem	0.06	2.71	<0.01
*R* ^2^	0.26		
*F*	46.92***		

N = 1377; Gender (1 = male, 2 = female); Grade (1 = freshman, 2 = sophomore, 3 = junior, 4 = senior); Only child (1 = only child, 2 = not only child); Residence (1 = cities, 2 = towns, 3 = villages). ***p < 0.001 (two-tailed).

**FIGURE 3:**
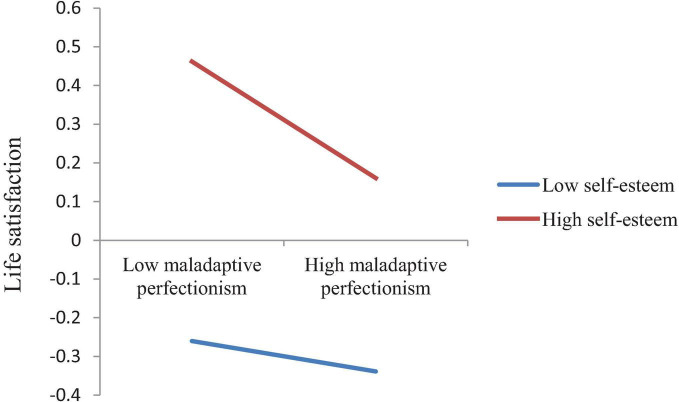
The interaction effect of maladaptive perfectionism and self-esteem on life satisfaction.

**FIGURE 4:**
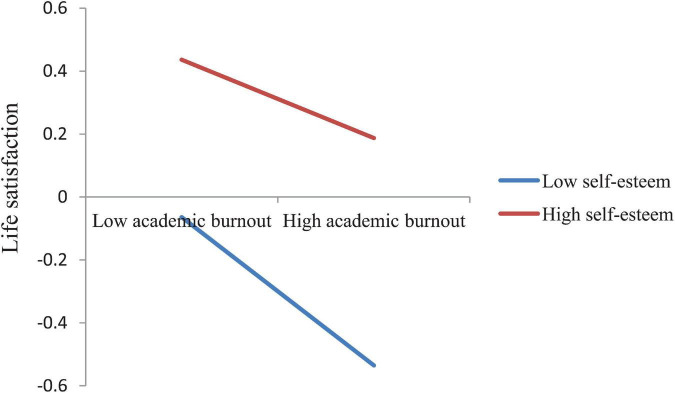
The interaction effect of academic burnout and self-esteem on life satisfaction.

In Model 2 with life satisfaction as the dependent variable, after controlling for socio-demographic variables (gender, age, grade, only child, residence), life satisfaction was significantly negatively associated with both maladaptive perfectionism (β = −0.10, *t* = −3.66, *p* < 0.001) and academic burnout (β = −0.18, *t* = −6.60, *p* < 0.001), while significantly positively related to self-esteem (β = 0.31, *t* = 10.90, *p* < 0.001). The interaction effect of maladaptive perfectionism and self-esteem (Maladaptive perfectionism × Self-esteem) on life satisfaction was significant (β = −0.06, *t* = −2.45, *p* < 0.05). The interaction effect of academic burnout and self-esteem (Academic burnout × Self-esteem) on life satisfaction was also significant (β = 0.06, *t* = 2.71, *p* < 0.01). We plotted these two interaction effects at two levels of self-esteem (1 SD below the mean and 1SD above the mean) in Figures 3, 4, respectively. Simple slope tests show that the association between maladaptive perfectionism and life satisfaction was stronger for medical students with high self-esteem (β*_*simple*_* = −0.15, *t* = −4.33, *p* < 0.001) than for medical students with low self-esteem (β*_*simple*_* = −0.04, *t* = −1.15, *p* > 0.05), while the association between academic burnout and life satisfaction was stronger for medical students with low self-esteem (β*_*simple*_* = −0.24, *t* = −6.69, *p* < 0.001) than for medical students with high self-esteem (β*_*simple*_* = −0.12, *t* = −3.78, *p* < 0.001).

In addition, the bias-corrected bootstrap analyses reveal that the indirect effect of maladaptive perfectionism on life satisfaction *via* academic burnout was moderated by self-esteem. Specifically, for medical students with high self-esteem (1SD above the mean), maladaptive perfectionism exerted a stronger effect on life satisfaction through the mediating effect of academic burnout [β = −0.026, SE = 0.009, 95%CI = (−0.047, −0.011)], while for medical students with low self-esteem (1SD below the mean), maladaptive perfectionism exerted a weaker effect on life satisfaction through the mediating effect of academic burnout [β = −0.019, SE = 0.009, 95%CI = (−0.038, −0.001)].

## Discussion

Our study used a large sample of undergraduate medical students to explore the associations between maladaptive perfectionism (a personality trait), life satisfaction (a subjective well-being indicator), academic burnout (a mental health problem) and self-esteem (a psychological resource). The results show that academic burnout played a significant mediating role in the relationship between maladaptive perfectionism and life satisfaction among medical students, and self-esteem moderated this mediation.

### The Mediating Role of Academic Burnout

Results of the mediation analysis show that academic burnout played a significant mediating role in the relationship between maladaptive perfectionism and life satisfaction among medical students. Higher levels of maladaptive perfectionism were associated with higher levels of academic burnout, which in turn, led to lower levels of life satisfaction. Conversely, lower levels of maladaptive perfectionism were associated with lower levels of academic burnout, which in turn, contributed to higher levels of life satisfaction. Students with high levels of maladaptive perfectionism are afraid of making mistakes, hesitant to take actions and shoulder pressure under high parental expectations (Frost et al., 1990), which make them more vulnerable to academic burnout. Students who suffered from academic burnout felt exhausted toward study, developed improper learning behaviors and gradually lost interest and enthusiasm in study. As a consequence, they performed poorly in academic work and the perceived academic inefficacy undermined their confidence and self-worth (Lian et al., 2005). These negative emotions and psychological maladjustment brought by academic burnout, could in turn, result in lower life satisfaction among medical students. Although previous research has demonstrated the positive correlation of maladaptive perfectionism with academic burnout (Yu et al., 2016), the negative association of academic burnout with life satisfaction (Wang et al., 2019), and the negative relation between maladaptive perfectionism and life satisfaction (Stoeber and Stoeber, 2009), to the best of our knowledge, no study has probed the mediating role of academic burnout in the relationship between maladaptive perfectionism and life satisfaction among medical students, and our study filled in this research gap.

### The Moderating Role of Self-Esteem

The moderated mediation analyses reveal that self-esteem moderated the mediating role of academic burnout in the relationship between maladaptive perfectionism and life satisfaction among medical students. Maladaptive perfectionism exerted a stronger effect on life satisfaction *via* the mediating role of academic burnout for students with high self-esteem than for students with low self-esteem. Medical students with high self-esteem levels were more protected from the positive impacts of maladaptive perfectionism on academic burnout, and from the negative impacts of maladaptive perfectionism on life satisfaction through the mediating effect of academic burnout.

Global self-esteem refers to how an individual evaluates his or her self-worth (Mruk, 2006) and is considered an important factor affecting a person’s psychological health (Rosenberg, 1965). Research shows that people with high self-esteem enhance initiatives that help them achieve life goals and bridge the gap between actual self and ideal self (Mruk, 2006). People with a healthy level of self-esteem do not worry excessively about the past but learn from it and live in the present, fully believing in their own capabilities to solve problems without hesitating after difficulties and failures (Bonet, 1997). Studies have shown that self-esteem can be seen as a source of resiliency, which promotes a coping mechanism that facilitates academic achievement (Crocker and Luhtanen, 2003); thus, students with high self-esteem tend to adopt problem-focused coping strategies rather than avoidance coping styles, and this approach helps them buffer the negative effects of maladaptive perfectionism had on life satisfaction *via* academic burnout.

Results of simple slope tests show that when self-esteem moderated the paths from maladaptive perfectionism to academic burnout and from maladaptive perfectionism to life satisfaction, the β value for high self-esteem (1SD above the mean) was larger than the β value for low self-esteem (1SD below the mean), as shown in Figure 2 and in Figure 3. However, when self-esteem moderated the path from academic burnout to life satisfaction, the situation was opposite, with the β value for low self-esteem larger than the β value for high self-esteem, as shown in Figure 4. These results indicate that maladaptive perfectionism and self-esteem together had a synergistic effect, i.e., medical students with high self-esteem were more affected by maladaptive perfectionism with regard to academic burnout and life satisfaction. In other words, if we want to lower medical students’ academic burnout and raise their life satisfaction, we need to simultaneously raise students’ self-esteem levels and reduce their maladaptive perfectionism levels.

### Implications and Limitations

The present study has important implications for medical education. Besides reaffirming the conclusion from previous research that maladaptive perfectionism was significantly negatively related to life satisfaction, the present study also clarified the mechanism underlying this relationship among medical students. Specifically, the present study demonstrated how maladaptive perfectionism affected medical students’ life satisfaction (i.e., through the mediator of academic burnout), and under what conditions maladaptive perfectionism affected medical students’ life satisfaction differently (i.e., through the moderator of self-esteem). Results of the present study show that maladaptive perfectionism exerted a negative impact on medical students’ life satisfaction through the mediating effect of academic burnout. This indicates that besides lowering students’ maladaptive perfectionism to raise their life satisfaction, we can also try to improve students’ life satisfaction levels through lowering their academic burnout levels. Psychological interventions such as implementing cognitive behavioral therapy (CBT) and mental health education courses in medical education have already been demonstrated to be effective in helping medical students reduce maladaptive perfectionism levels, decrease academic burnout levels and increase life satisfaction levels (Chand et al., 2018; Wang and Du, 2020). In addition, two recent cross-sectional studies by Khosravi and Khosravi et al. found that academic burnout was significantly associated with physical activities in medical students and encouraging medical students to do more physical exercise may lower their academic burnout levels (Khosravi et al., 2020; Khosravi, 2021). In this regard, interventional studies need to be conducted in the future to examine the effectiveness of physical activities in helping medical students reduce academic burnout.

As the moderated mediation model shows that compared with students with low self-esteem levels, students with high self-esteem levels were more protected from the negative impacts of maladaptive perfectionism and academic burnout on their life satisfaction levels, medical institutions may consider implementing self-esteem promotion programs to help medical students buffer against the detrimental effect of maladaptive perfectionism on life satisfaction *via* the mediator of academic burnout. Some studies have already demonstrated the effectiveness of measures such as psychosocial interventions, character strengths-based interventions, exercise interventions, cognitive behavioral therapy (CBT) and Eye Movement Desensitization and Reprocessing therapy (EMDR) in helping different population groups raise self-esteem levels (Gothe et al., 2011; Rebecca et al., 2016; Yeun and Han, 2016; Griffioen et al., 2017; Kolubinski et al., 2018). However, we found that research on effective interventions to help medical students enhance self-esteem is scarce and the present study prompts the urgent need for more research in this field.

Several limitations of the present study should be acknowledged. First, the cross-sectional design did not allow conclusions about causality to be drawn. Second, the research was conducted at only two medical universities in China, so generalizations should be made with caution. Third, response bias cannot be avoided since we used self-administered questionnaires as the data collection method. Fourth, in the present study, academic burnout was found to play a partial mediating role in the relationship between maladaptive perfectionism and life satisfaction among medical students, which indicated that there are other mediators in this relationship. Thus, it is recommended that future studies further explore the mechanism underlying the relationship between maladaptive perfectionism and life satisfaction among medical students, which can shed light on developing effective interventions to improve medical students’ psychological health.

## Conclusion

The present study found that academic burnout partially mediated the association of maladaptive perfectionism with life satisfaction in medical students and self-esteem moderated this mediation. Maladaptive perfectionism not only had a significantly detrimental impact on life satisfaction, but also exerted a negative impact on life satisfaction through the mediating effect of academic burnout. Compared with medical students with low self-esteem, students with high self-esteem were more protected from the negative impact of maladaptive perfectionism on life satisfaction *via* the mediator of academic burnout. These findings indicate that medical educators need to conduct more research on identifying effective interventions to reduce medical students’ levels of maladaptive perfectionism and academic burnout, and enhance self-esteem levels in order to promote their life satisfaction.

## Data Availability Statement

The original contributions presented in the study are included in the article/supplementary material, further inquiries can be directed to the corresponding author.

## Ethics Statement

The studies involving human participants were reviewed and approved by the Institutional Review Board of China Medical University. The patients/participants provided their informed consent form online to participate in this study.

## Author Contributions

QW was in charge of the study design, questionnaire survey, and drafting and revising the manuscript. HW made substantial intellectual contributions to the conception of the study and analyses of the data and draft of the manuscript. Both authors read and approved the final version of the manuscript.

## Conflict of Interest

The authors declare that the research was conducted in the absence of any commercial or financial relationships that could be construed as a potential conflict of interest.

## Publisher’s Note

All claims expressed in this article are solely those of the authors and do not necessarily represent those of their affiliated organizations, or those of the publisher, the editors and the reviewers. Any product that may be evaluated in this article, or claim that may be made by its manufacturer, is not guaranteed or endorsed by the publisher.
